# Clinical study on endovascular recanalization of non-acute symptomatic middle cerebral artery occlusion

**DOI:** 10.3389/fneur.2022.1036661

**Published:** 2023-01-09

**Authors:** Jinchao Xia, Hao Li, Kun Zhang, Ziliang Wang, Tianxiao Li

**Affiliations:** Department of Cerebrovascular Disease, Henan Provincial People's Hospital, Zhengzhou University People's Hospital, Zhengzhou, Henan, China

**Keywords:** endovascular, recanalization, anterior circulation, symptomatic, middle cerebral artery, non-acute phase

## Abstract

**Objective:**

Endovascular recanalization in patients with non-acute symptomatic middle cerebral artery occlusion remains clinically challenging. Here, we aimed to evaluate the feasibility and safety of endovascular recanalization for non-acute symptomatic middle cerebral artery occlusion and propose a new patient classification.

**Methods:**

Between January 2019 and December 2021, 88 patients with non-acute symptomatic middle cerebral artery occlusion underwent prospective endovascular recanalization at our hospital. All patients were divided into three groups according to occlusion length, occlusion duration, occlusion nature, calcification of the occlusion site, and occlusion angulation. The indicators of each group were analyzed, including general baseline data, imaging data, surgical conditions, and follow-up results.

**Results:**

Of the 88 patients, 73 were successfully recanalized and 15 were abandoned because the instruments either could not reach the distal true lumen of the occlusion or broke through the blood vessels. The overall technical success rate was 83.0% (73/88), and perioperative complications occurred in 15 patients. Preoperatively, all patients were divided into three risk groups: low, medium, and high. From the low- to high-risk groups, the recanalization rate gradually decreased (100.0, 91.7, and 16.7%, respectively, *P* = 0.020), the perioperative complication rate gradually increased (0, 13.9, and 83.3%, respectively, *P* < 0.001), the proportion of the modified Rankin scale scores >2 at 90 days increased (0, 11.7, and 50.0%, *P* < 0.001), and the restenosis/reocclusion rates in the 73 cases of successful recanalization increased (0, 16.1, and 100%, *P* = 0.012) during follow-up.

**Conclusion:**

Endovascular recanalization may be feasible and safe in well-selected patients with non-acute symptomatic middle cerebral artery occlusion, especially in the low- and medium-risk groups.

## 1. Introduction

Middle cerebral artery occlusion (MCAO) is devastating and associated with high morbidity and mortality rates. The optimal treatment for non-acute MCAO remains unclear. Despite optimal medical therapy, such patients are still at high risk of recurrent ischemic stroke (1). Although some patients do not have symptoms of cerebral ischemia, long-term cerebral hypoperfusion can lead to brain atrophy, which can manifest as cognitive impairment, especially for non-acute MCAO in the dominant hemisphere (2). Currently, there is no effective treatment for non-acute symptomatic MCAO, and previous superficial temporal artery–middle cerebral artery bypass surgery has been confirmed with little to no benefit (3, 4). In recent years, endovascular techniques (ETs) have been shown to be an effective treatment for patients with acute ischemic stroke within 6 h, rescuing ischemic brain tissue and leading to good patient prognosis (5, 6). Some recent studies using ETs to treat chronic internal carotid artery occlusion (ICAO) achieved satisfactory results (7, 8). However, the heterogeneity of perioperative outcomes and the high rate of complications prompted us to explore which patients are the best candidates and perform individualized risk stratification. In this study, we propose a classification of patients for preoperative assessment based on preoperative imaging, including the length of occlusion, occlusion duration, nature of occlusion, calcification at the occlusion site, and angulation of the occlusion segment. The patients were scored and classified to evaluate the technical risk of endovascular recanalization surgery, explore the feasibility and safety of ETs for non-acute symptomatic MCAO, and provide a reference for patient selection and risk stratification.

## 2. Materials and methods

### 2.1. Patient selection

A total of 88 patients with non-acute symptomatic MCAO (M1 segment) were admitted to our hospital from January 2019 to December 2021, according to the patient's imaging examination, occlusion length, duration, and nature, calcification of the occlusion site, and angulation of the occluded segments (Table 1). All patients were divided into three groups: low-risk (0–2 points), medium-risk (3–4 points), and high-risk (5–7 points). Occlusion length was defined as the distance between the proximal end of the occlusion and the visible distal segment on preoperative imaging (computed tomography angiography [CTA] or digitally subtracted angiography [DSA]). Occlusion duration was defined as 100% occlusion of the vascular lumen cross-section on CTA or magnetic resonance angiography (MRA), with the occlusions confirmed by DSA and the duration from the date of initial diagnosis to the day of surgery determined by a three-person expert group consultation. The occlusion nature was determined according to the patient's past history of atherosclerosis risk factors and preoperative examination to confirm the diagnosis. Occlusion calcification was defined as “any form of calcified lesions within the outline of the blood vessel” on CT and determined by a three-person expert group consultation. Occlusion angulation was defined as the included angle of the axial extension line of the occlusion distal and proximal vessels; if the occluded end was flush or the segment was too short to determine the axial direction, the occlusion angle of the blood vessel was compared with the normal side. Informed consent was obtained from all the patients, and the study was approved by the institutional review board. The inclusion criteria were as follows: (1) MCAO diagnosed by CTA or MRA and confirmed using DSA; (2) occlusion duration >12 days, defined as the time from the date of initial diagnosis to the day of surgery; and (3) recurrent transient ischemic attack or stroke associated with MCAO despite optimal medical treatment. The exclusion criteria were as follows: (1) intracranial aneurysm and any bleeding disorder; (2) life expectancy <1 year due to other medical conditions; (3) large infarct core (ASPECT score <6 points); and (4) occlusion of the middle cerebral artery that continued to the internal carotid artery bifurcation without a stump.

**Table 1:** Scores for successful recanalization of non-acute symptomatic MCAO.

**Variables**	**Score**
**Length of occlusion**	
≤5 mm	0
5–10 mm	1
≥10 mm	2
**Occlusion duration**	
≤1 month	0
1–3 months	1
>3 months	2
**Occlusion calcified**	
No	0
Yes	1
**Occlusion nature**	
Atherosclerotic	0
Others^*^	1
**Occlusion angulation**	
Straight	0
Angulation ≤ 30°	1
Angulation >30°	2
**Total score**	
0–2	Low risk
3–5	Medium risk
6–8	High risk

MCAO, middle cerebral artery occlusion;

* indicates occlusion caused by inflammation, dissection, radioactive vascular occlusion, or fibromuscular dysplasia.

### 2.2. Operative method

All procedures were performed by neurointerventional doctors with the patients under general anesthesia. After placement of the sheath introducer, heparin was administered intravenously to maintain an activated clotting time of 200–300 s. The 6F guiding catheter was advanced into the internal carotid artery, and the micro-guide wire cooperated with the microcatheter to reach the distal true lumen through the occluded segment. The angioplasty balloon catheter ascended along the exchange micro-guide wire, passed through the occluded lesion for expansion (the balloon size was selected according to the diameter of the proximal normal middle cerebral artery), and inflated to 6 atm for 30–60 s. If dissection or slow blood flow occurred after angioplasty, stent implantation was performed, mainly using the LVIS stent (MicroVention, USA), Neuroform EZ stent (Stryker Neurovascular, USA), or Enterprise stent (Codman, USA), with the choice of stents depending on the judgment of the operator. Successful recanalization was defined as modified thrombolysis in cerebral infarction score (mTICI) of ≥2b and residual stenosis of <50%.

### 2.3. Surgical management

Combination therapy with oral aspirin (100 mg) and clopidogrel (75 mg) was initiated at least 3 days before endovascular surgery. Thromboelastography was used to assess platelet reactivity. Aspirin resistance was defined as arachidonic acid-induced platelet aggregation inhibition <50%, and clopidogrel resistance was defined as <30% inhibition of ADP-induced platelet aggregation. None of the patients were aspirin-resistant, while 12 patients were resistant to clopidogrel and were treated instead with ticagrelor 90 mg twice daily. Dual antiplatelet therapy was maintained for 3–6 months, followed by lifelong maintenance of aspirin or clopidogrel monotherapy.

### 2.4. Clinical follow-up

Angiography and clinical follow-up were performed 3–6 months after surgery to assess the patency status of recanalized vessels, such as recurrence of stenosis, which should be measured by two neurointerventional specialists, according to the WASID criteria (9). Clinical follow-up mainly identified whether the patient had neurological deficits. If present, evaluation was conducted to determine whether it was related to the patient's surgery, stenting, or recanalization, and CT or magnetic resonance imaging (MRI) examination was performed. The initial and final angiography results were intervened by two neurointerventional experts to reach a consensus. In-stent restenosis was defined as angiographically proven in-stent or within 5 mm of the stent margin, with stenosis >50% and lumen diameter loss of 20% immediately after surgery. The mRS scores were divided into good (mRS, 0–2), moderate (mRS, 3), and poor (mRS, 4–5).

### 2.5. Statistical methods

Normally distributed quantitative variables are presented as mean ± standard deviation for all continuous variables, non-normally distributed variables are presented as a median and interquartile range, and categorical variables are presented as numbers and proportions. Comparisons between groups were performed using the Kruskal–Wallis test for continuous variables or the approximate chi-square test for categorical variables. The chi-square test was used for comparisons between the three groups, and differences were considered statistically significant when *P* < 0.05. All statistical analyses were performed using SPSS 22.0.

## 3. Results

### 3.1. Baseline characteristics and overall results

From January 2019 to December 2021, 88 consecutive patients underwent endovascular recanalization therapy for non-acute symptomatic MCAO. Table 2 summarizes the patients' baseline data. Of the 88 patients, 54 were men and 34 were women, with ages ranging from 31 to 80 years (mean, 57.0 ± 10.2 years). All patients had a history of ischemic stroke or TIA. The median duration from imaging diagnosis of occlusion to the day of recanalization surgery was 37 days (interquartile range 25–55). The overall technical success rate was 83.0% (73/88). The perioperative complication rate was 17.0% (15/88), with postoperative complications in 15 cases, of which three cases were asymptomatic, mild ischemic stroke occurred in six cases (National Institutes of Health Stroke Scale [NIHSS] score ≤4), three cases suffered an intracerebral hemorrhage, subarachnoid hemorrhage occurred in two cases, and one patient died from cardiac arrest. Stroke or death within 30 days occurred in 2.3% (2/88) of patients. Out of 88 patients, 84 (four patients lost to follow-up) had a mean clinical follow-up time of 4.52 ± 1.29 months. Stroke or mortality after 30 days occurred in 6.0% (5/84) of patients, with one death due to intracerebral hemorrhage, severe ischemic strokes in two patients (NIHSS score was 15, and modified Rankin scale [mRS] score was 5), and mild ischemic strokes in two patients (NIHSS score ≤4). The mean angiographic follow-up time of 75 patients was 4.79 ± 1.48 months, with four in-stent restenosis and three reocclusions.

**Table 2:** Baseline characteristics of 88 patients with MCAO.

**Variables**	**Total (*n* = 88)**	**Low-risk group (*n* = 40)**	**Medium-risk group (*n* = 34)**	**High-risk group (*n* = 14)**	**χ^2^/F**	***P***
F/M	34/54	11/29	17/17	6/8	0.972	0.615
Age (years)	57.0 ± 10.2	56.3 ± 9.7	58.9 ± 10.2	54.4 ± 11.6	1.150	0.322
Height (cm)	166.3 ± 8.8	167.1 ± 8.5	165.3 ± 9.7	166.4 ± 7.7	0.384	0.683
Weight (kg)	71.8 ± 11.5	72.0 ± 10.0	70.6 ± 12.5	73.9 ± 13.5	0.411	0.664
BMI	26.1 ± 3.9	25.9 ± 3.6	26.2 ± 4.1	26.6 ± 4.3	0.182	0.834
Infarction/TIA	77/11	35/5	31/3	11/3	1.073	0.585
Hypertension	49 (55.7%)	20 (50.0%)	21 (61.8%)	8 (57.1%)	0.298	0.862
Diabetes mellitus	32 (36.4%)	17 (42.5%)	13 (38.2%)	2 (14.3%)	1.956	0.367
Hyperlipidemia	25 (28.4%)	11 (27.5%)	11 (32.4%)	3 (21.4%)	0.348	0.840
Alcohol consumption	33 (37.5%)	19 (47.5%)	10 (29.4%)	4 (28.6%)	1.413	0.493
Smoking	34 (37.5%)	19 (47.5%)	11 (32.4%)	4 (28.6%)	1.099	0.577
Presurgery mRS					43.892	0.062
0	56 (63.6%)	21 (52.5%)	24 (70.6%)	11 (78.6%)		
1	25 (28.4%)	14 (35.0%)	8 (23.5%)	3 (21.4%)		
2	7 (8.0%)	5 (12.5%)	2 (5.9%)	0 (0)		
3	0 (0)	0 (0)	0 (0)	0 (0)		
4	0 (0)	0 (0)	0 (0)	0 (0)		
5	0 (0)	0 (0)	0 (0)	0 (0)		

MCAO, middle cerebral artery occlusion; F/M, female/male; BMI, body mass index; TIA, transient ischemic attack; mRS, modified Rankin scale.

### 3.2. Subgroup analysis

**Figure 1:**
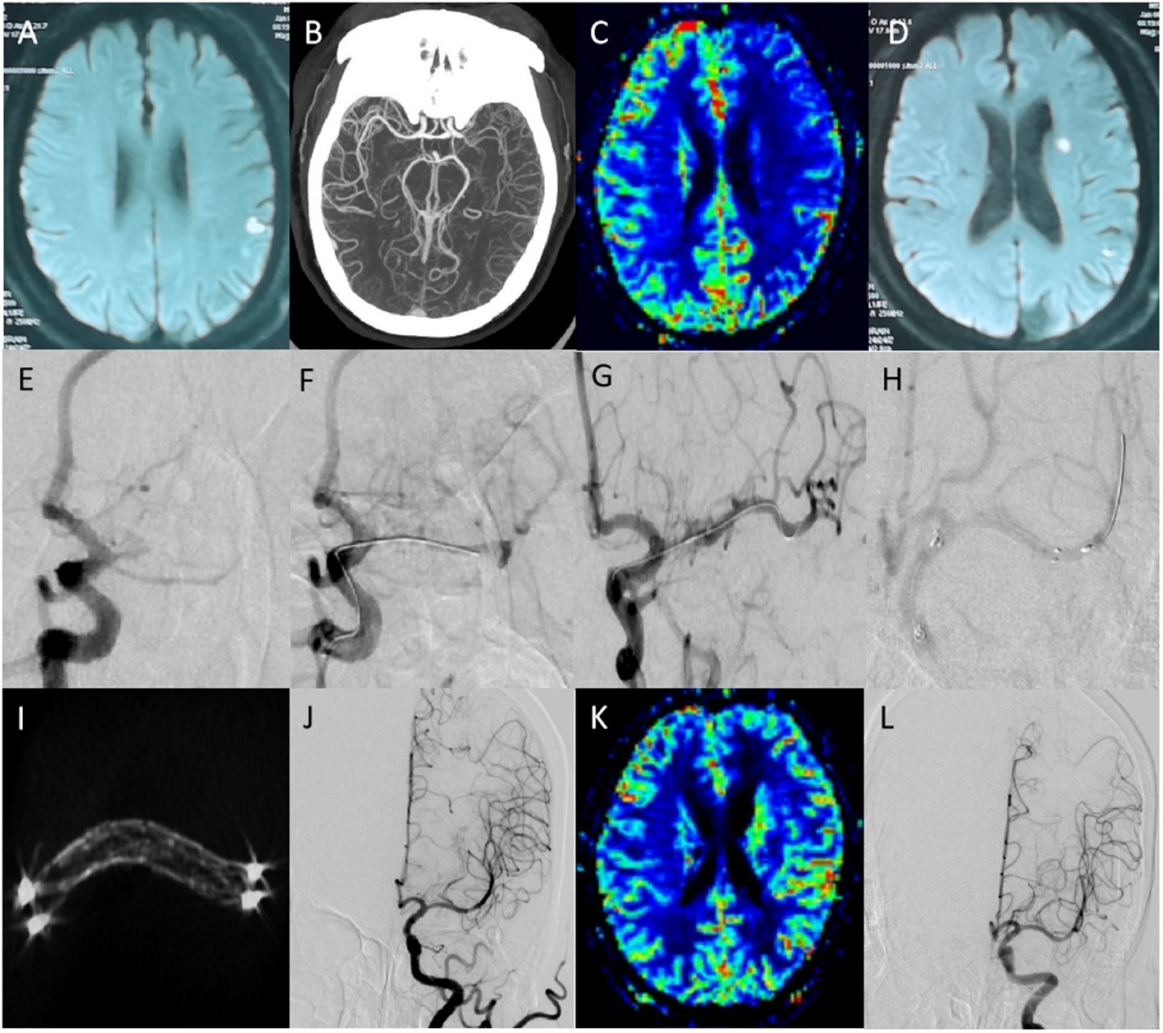
Clinical data of a low-risk patient. Male patient, 65 years old; chief complaint: right limb weakness with slurred speech for 21 days, relapsed for 3 days; past medical history: hypertension for 6 years; physical examination: the right limb muscle strength was grade 3, and the muscle tone was normal; the mRS score was 2. **(A)** Preoperative magnetic resonance imaging (MRI) shows acute cerebral infarction in the left cerebral parietal lobe. **(B)** Computed tomography angiography shows the M1 segment occlusion of the left middle cerebral artery. **(C)** Preoperative perfusion-weighted imaging (PWI) shows left cerebral hypoperfusion. **(D)** MRI after symptom recurrence shows left basal ganglia and parietal lobe acute cerebral infarction. **(E)** Preoperative digitally subtracted angiography (DSA) shows the left middle cerebral artery occlusion (occlusion length <5 mm, occlusion duration <3 months, atherosclerotic, no calcification, straight). **(F)** The micro-guide wire passed through the occluded segment during the operation. **(G)** The Gateway 2.0 × 10 mm balloon was used for accurate positioning and 4ATM expansion. **(H)** Neuroform 3.5 × 20 mm was ascended along the stent catheter, accurately positioned, and released. **(I)** The reconstructed stent is visible, and the stent is well expanded. **(J)** Postoperative anteroposterior angiography. **(K)** PWI shows that the bilateral perfusion was symmetrical, and the left cerebral perfusion was significantly improved. **(L)** Angiography was repeated 5 months later, which showed stent patency, and the mRS score was 0.

Table 3 summarizes the clinical data of the 88 patients. From the low- to high-risk groups, the recanalization rate gradually decreased (100.0, 91.7, and 16.7%, respectively, *P* = 0.020), the perioperative complication rate gradually increased (0, 13.9, and 83.3%, respectively, *P* < 0.001), the proportion of mRS scores >2 at 90 days increased (0, 11.7, and 50.0%, respectively, *P* < 0.001), and the restenosis/reocclusion rates in the 73 cases of successful recanalization increased (0, 16.1, and 100%, respectively, *P* = 0.012) during follow-up. Figure 1 shows the clinical data of a low-risk patient.

**Table 3:** Complications and clinical outcomes of 88 patients with MCAO.

**Variables**	**Total (*n* = 88)**	**Low-risk group (*n* = 40)**	**Medium-risk group (*n* = 34)**	**High-risk group (*n* = 14)**	**χ2/F**	***P***
Recanalization rate	83.0% (73/88)	100% (40/40)	91.2% (31/34)	14.3% (2/14)	7.809	0.020
Treatment methods					0.252	0.778
BD only	10 (11.4%)	5 (12.5%)	5 (14.7%)	0 (0)		
BD+SE stent	63 (71.6%)	35 (87.5%)	26 (76.5%)	2 (14.3%)		
TICI grade: IIb/III	26/47	13/27	12/19	1/1	0.220	0.896
Complication rate	15 (17.0%)	0 (0)	4 (11.8%)	11 (78.6%)	24.727	0.000
Intracranial bleeding	5 (5.7%)	0 (0)	2 (5.9%)	3 (21.4%)		
Brain infarction	9 (10.2%)	0 (0)	2 (5.9%)	7 (50.0%)		
Cardiac arrest	1 (1.1%)	0 (0)	0	1 (7.1%)		
Follow-up (months)	4.5 ± 1.3	4.7 ± 1.3	4.3 ± 1.3	4.7 ± 1.3	1.031	0.361
90-day mRS					26.132	0.000
0	61 (69.3%)	37 (92.5%)	21 (61.8%)	3 (21.4%)		
1	9 (10.2%)	3 (7.5%)	6 (17.6%)	0 (0%)		
2	3 (3.4%)	0 (0)	2 (5.9%)	1 (7.1%)		
3	5 (5.7%)	0 (0)	2 (5.9%)	3 (21.4%)		
4	5 (5.7%)	0 (0)	2 (5.9%)	3 (21.4%)		
5	1 (1.1%)	0 (0)	0	1 (7.1%)		
Restenosis	4 (5.5%)	0 (0)	3 (9.7%)	1 (50.0%)	7.924	0.019
Reocclusion	3 (4.1%)	0 (0)	2 (6.5%)	1 (50.0%)	8.865	0.012

MCAO, middle cerebral artery occlusion; BD, balloon dilatation; SE stent, self-expanding stent implantation; TICI, cerebral infarction thrombolysis; mRS, modified Rankin scale.

## 4. Discussion

Non-acute MCAO is a chronic vascular occlusion, which can be caused by atherosclerosis. It is the end result of the natural development of atherosclerosis. The continuous enlargement of the local atherosclerotic plaque or the formation of a new thrombus based on atherosclerotic plaque leads to vascular occlusion (10, 11). Non-acute MCAO is a highly heterogeneous disease, and its clinical manifestations and prognosis vary greatly among individuals owing to the degree of cerebrovascular collateral compensation (12, 13). The collateral circulation is well compensated, and patients may not have any clinical symptoms. Patients with poor collateral circulation compensation may experience repeated events such as cerebral infarction/TIA; therefore, active intervention measures for the non-acute phase occlusion of the middle cerebral artery with poor collateral circulation compensation have gradually been recognized (14). In recent years, many medical centers have attempted endovascular recanalization therapy for patients with non-acute symptomatic large vessel occlusion based on the experience of intravascular recanalization in acute intracranial large vessel occlusion (15–18). Patients with large intracranial artery occlusions are selected to undergo endovascular recanalization, which can help prevent repeated cerebrovascular ischemia events and promote functional recovery in patients with disabilities (17, 18). It is critical to select patients who could benefit from revascularization therapy. Previous studies on endovascular recanalization for non-acute symptomatic intracranial large artery occlusion have shown that patients with clinical symptoms caused by hypoperfusion can benefit most from endovascular recanalization therapy (19). In this study, only patients with non-acute MCAO with poorly compensated collateral circulation and a poor response to medical drug treatment were selected for endovascular recanalization. Such patients have hemodynamic disorders in their pathogenesis and theoretically obtain the most potential benefit from endovascular recanalization therapy. In this study, the low-risk group patients with a recanalization rate of 100% and a low risk of perioperative complications (0%) were identified as the best candidates for endovascular recanalization treatment. To the best of our knowledge, this study represents the largest serial case study on endovascular recanalization treatment in patients with non-acute symptomatic MCAO.

Whether the micro-guide wire can pass through the occlusion segment smoothly is the key to the success of the recanalization operation, but it is related to the occlusion length, duration, nature, calcification of the occlusion site, and occlusion angulation. The longer the occlusion length, the more difficult it is to pass. This study demonstrated that the technical challenges of occlusion and patency are related to the occlusion length, with the low-risk group characterized by shorter lesions having the highest recanalization rates, and in the high-risk group, an occlusion length of >10 mm was one of the factors leading to failure of recanalization. Also, the more severe the angulation of the occlusion segment, the greater the possibility of vessel perforation during the exploration of the micro-guide wire, which is related to the difficulty in identifying true vessels when crossing the occlusion. Furthermore, the longer the occlusion duration, the more severe the calcification or fibrosis of the occluded segment and the more difficult the recanalization process will be. Coronary recanalization experience in chronic occlusion also shows that when the occlusion nature is atherosclerotic and the occlusion duration is <3 months, the successful recanalization rate is high; however, when the occlusion length is >5 mm, calcification and angulation of the occluded segment are severe, and the recanalization rate is low (20–22). In this study, all patients were divided into three groups according to occlusion length, duration, and nature, calcification of the occlusion site, and angulation of the occluded segments. From the low- to high-risk groups, the recanalization rate gradually decreased, whereas the complication rate, 90-day mRS score, and follow-up rate of restenosis/reocclusion in patients with successful recanalization increased gradually. In addition, in the low- and medium-risk groups, recanalization rates (100 and 91.7%, respectively) and perioperative complication rates (0 and 13.9%, respectively) were acceptable; hence, patients categorized in these groups should be considered the best candidates for endovascular recanalization therapy.

In 1998, Mori et al. proposed a cerebral arteriography classification that classifies lesions according to the length and geometry of intracranial atherosclerotic stenosis (23). However, unlike stent placement surgery for stenosis, the anatomical direction and course of intracranial occlusions cannot be determined through imaging. Therefore, endovascular recanalization surgery in chronic total occlusion was more dependent on the surgeon's experience. For surgeons, the length of occlusion judged by angiography is mainly based on the distance between the proximal occlusion and distal collateral vessel reconstruction, which may be longer than the true length of the underlying atherosclerotic lesion. Preoperative high-resolution MRI, the original image of CTA, or MRA can also help to determine the shape and length of the occluded segment. When the surgeon tries to pass the micro-guide wire through the occlusion segment at an invisible angle, attention should be paid to the deviation of the axial direction of the blood vessel; otherwise, the blood vessel would perforate and rupture. In this study, we used the ipsilateral advanced arterial image as a road map to simultaneously display the distal and proximal ends of the occluded vessel so that the operator can more intuitively observe the direction of the occluded vessel and predictably manipulate the guide wire; this approach reduced complications and increased the success rate of recanalization.

There were some limitations to our study. First, the study was a single-center study and relatively small sample size. Second, this study was non-randomized controlled trials and lack of quantitative perfusion studies comparing regional blood volumes. Third, patient's heterogeneity and Chinese patients enrolled only may produce some bias to affect the generalization of the study. Finally, the lack of long-term follow-up data may limit the assessment of the overall restenosis rates.

In conclusion, endovascular recanalization may be feasible and safe in carefully selected patients with non-acute symptomatic MCAO, and it represents a potential alternative treatment approach, especially in low- and medium-risk groups.

## Data availability statement

The raw data supporting the conclusions of this article will be made available by the authors, without undue reservation.

## Ethics statement

The studies involving human participants were reviewed and approved by the Ethics Committee of Zhengzhou University People's hospital. The patients/participants provided their written informed consent to participate in this study. Written informed consent was obtained from the individual(s) for the publication of any potentially identifiable images or data included in this article.

## Author contributions

JX established the study idea and designed the manuscript structure. KZ and HL were responsible for the data collection. ZW analyzed the data and wrote the manuscript. TL modified and revised the manuscript. All authors have read and approved the final version of the manuscript.
